# Post-TPS needle placement planning for robotic-assisted LDR seed brachytherapy

**DOI:** 10.3389/fonc.2026.1730072

**Published:** 2026-02-11

**Authors:** Yilun Fan, Xuehe Zhang, Lichen Liu, Changle Li, Chong Yao, Jie Zhao

**Affiliations:** 1 State Key Laboratory of Robotics and System, Harbin Institute of Technology, Harbin, Heilongjiang, China; 2 Harbin Institute of Technology Hospital, Harbin Institute of Technology, Harbin, Heilongjiang, China

**Keywords:** Hough transform, LDR brachytherapy, optimization, robot surgery, treatment planning

## Abstract

**Introduction:**

Low-dose-rate (LDR) seed brachytherapy treatment planning systems (TPSs) can generate dose-optimized seed distributions; however, translating these plans into robot-executable needle insertions introduces additional geometric, anatomical, and robotic constraints that may limit clinical feasibility.

**Methods:**

This study presents an optimization framework for robotic-assisted LDR seed brachytherapy that refines needle trajectories based on preplanned seed distributions. The framework explicitly incorporates anatomical safety constraints and robotic feasibility requirements while aiming to preserve the original dosimetric quality.

**Results:**

Simulation studies using thoracic and upper abdominal anatomical models show that the proposed method can generate clinically executable needle placements with minimal deviation from the planned seed positions. Phantom experiments further demonstrate the practical feasibility and placement accuracy of the optimized trajectories under robotic operating conditions.

**Discussion:**

The proposed framework improves the feasibility and reproducibility of robotic-assisted LDR seed implantation in thoracic and related anatomical settings, offering a practical pathway toward safer and more reliable clinical deployment.

## 1 Introduction

Brachytherapy (BT) is a long-established modality for the treatment of malignant tumors, with a clinical history spanning more than a century. By implanting radioactive sources directly into or near the target volume, BT exploits the inverse-square law of radiation dose distribution to achieve highly localized dose delivery while sparing surrounding healthy tissues. Among its variants, low-dose-rate (LDR) seed brachytherapy has been widely applied in the treatment of prostate, breast, head and neck, and gynecological malignancies, as well as selected thoracic and abdominal tumors, including liver metastases, with well-documented clinical efficacy (1, 2).

The widespread use of robotic-assisted surgical techniques has improved the accuracy (3, 4) and safety of brachytherapy while avoiding radiation exposure (1). The use of collaborative robots enables the planning of needle orientation and rotation, thus enhancing dose optimization (5). Brachytherapy surgical robots are becoming a popular and trending area of research. Traditional surgery requires a grid template for needle guidance. In the latest research, customized three-dimensional (3D)-printed non-coplanar templates have been shown to improve seed consistency (6, 7). Current surgical robot systems are gradually moving away from dependence on physical templates. One of the bottlenecks of using six-degree-of-freedom general-purpose collaborative robots for brachytherapy is that the surgical robot system needs to incorporate a treatment planning system (TPS) (5). Existing TPSs mainly focus on traditional coplanar needle placements. To the best of our knowledge, no TPS studies have considered multi-directional needle placement planning for BT.

LDR brachytherapy planning algorithms have been extensively studied for decades. In conventional grid template-based brachytherapy planning research, planners aim to deliver prescribed doses to control points in discrete target organs while minimizing doses delivered to organs at risk. The researchers use a binary selection array to represent choices among potential seeds determined by the template, modeling target functions and constraints of clinical requirements, making needle placement planning a mixed-integer planning (MIP) problem. This MIP can be solved using various methods such as branch and bound (8–11), simulated annealing (12, 13), genetic algorithm (GA) (14, 15), Monte Carlo (16), and greedy heuristic (11, 17, 18), as well as more cutting-edge methods based on compressed sensing models (19), knowledge-based models (20), and treatment planning with Generative Adversarial Networks (TP-GAN) (21). More advanced treatment planning algorithms are now considering non-coplanar templates (22–24).

Related planning challenges have also been investigated in other percutaneous interventions, such as radiofrequency ablation (RFA), where optimal needle or probe placement under geometric and anatomical constraints is similarly formulated as a MIP or continuous optimization problem (25, 26). Although these methods are not directly designed for brachytherapy, they provide valuable methodological insights into constrained needle trajectory planning in complex anatomical environments.

In most existing brachytherapy planning approaches, needle trajectories are optimized jointly with dosimetric objectives by directly evaluating dose–volume histogram (DVH) metrics such as V100, V150, and D90. This process often requires careful tuning of objective weights or selection among Pareto-optimal solutions, which can introduce manual intervention and limit reproducibility. In contrast, recent advances in dose optimization—using physics-based dose engines or learning-assisted planning tools—have demonstrated the ability to generate high-quality seed distributions that satisfy multiple clinical metrics prior to needle trajectory consideration (15, 27).

Motivated by these developments, this paper focuses on a post-planning needle trajectory optimization problem. In this work, we explicitly adopt a post-TPS formulation, where dosimetric trade-offs are assumed to be resolved upstream by the treatment planning system, and the proposed method focuses on translating a clinically acceptable seed distribution into robot-feasible needle trajectories without reintroducing dose-related hyperparameter tuning. Specifically, given a set of seed dwell positions optimized for dosimetric quality, we aim to determine robot-feasible needle paths that minimize seed displacement while satisfying geometric and robotic constraints. The proposed method is designed to address the practical feasibility of needle placement under arbitrary seed distributions, rather than to replace or re-optimize the underlying dose plan.

Simulation studies and phantom experiments are conducted in thoracic and abdominal anatomical settings to evaluate the proposed framework under realistic robotic constraints. The quality of the seed distribution itself is assumed to be clinically acceptable and is therefore beyond the scope of this study.

The remainder of this paper is organized as follows. Section 2 describes the planning framework and optimization method. Section 3 presents simulation and phantom experiments. Section 4 discusses results, limitations, and clinical implications.

## 2 Methods

**Figure 1:**
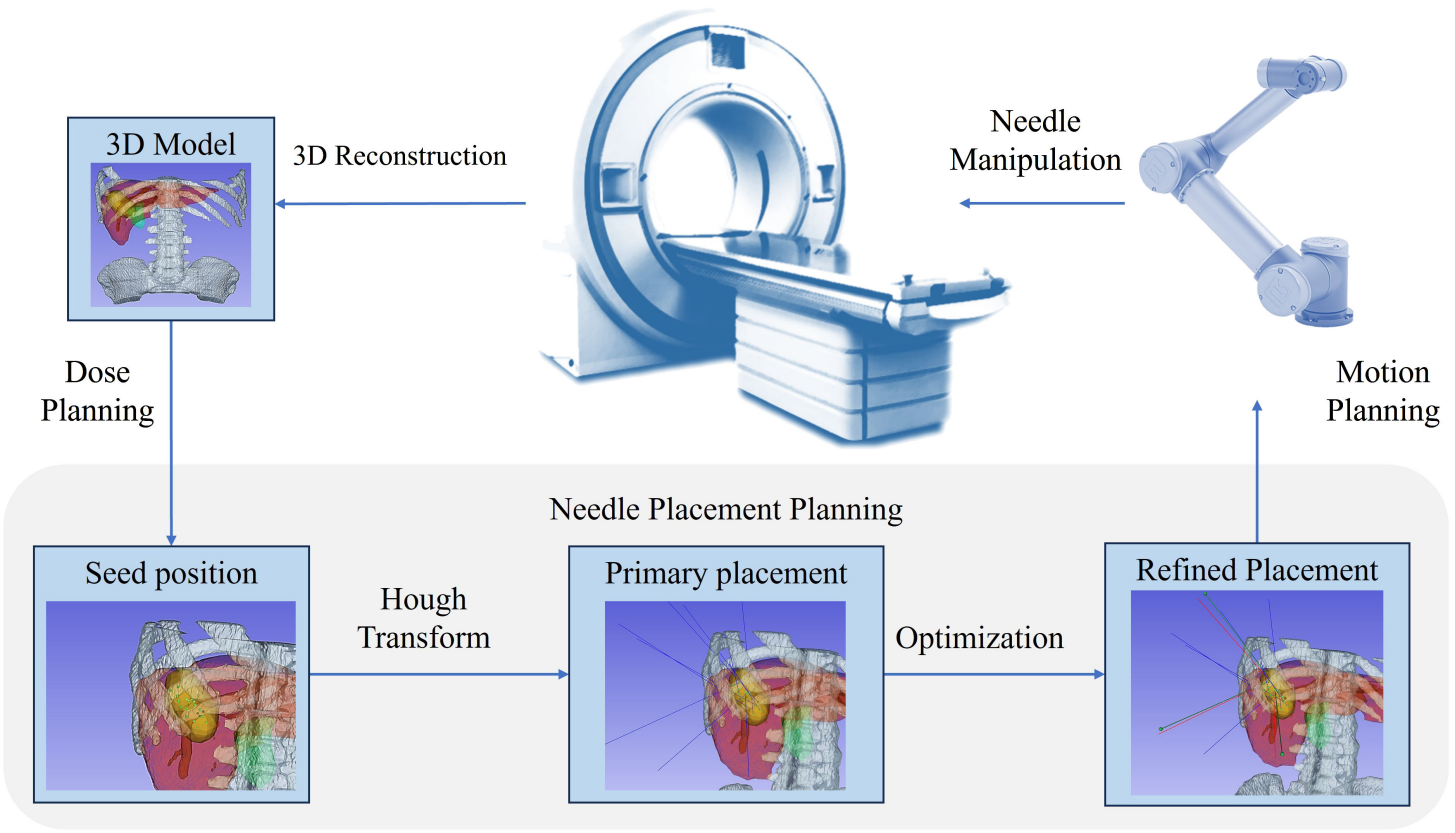
Brachytherapy surgery diagram.

The brachytherapy surgery diagram is shown in **Figure 1**. The process begins with the generation of a 3D reconstruction of the abdomen using CT images. Dose planning generates a set of seed dwell positions with optimal dosimetric metrics. The seeds are then used to determine needle placements. The robot performs motion planning according to the needle placement plan, guiding the end-effector to the planned insertion pose and advancing the needle to the target position. The subsequent seed implantation along the needle track is performed manually by the operator.

Multi-needle placement planning aims to determine a set of needle placements *t*_1_, *t*_2_, …, *t_n_* passing through all seeds *s*_1_, *s*_2_, …, *s_m_*, while adhering to surgical paradigms and robotic operational constraints. However, solving this problem by brute force is impractical due to the computational challenges involved, particularly when the number of seeds exceeds 50. Instead, we adopted a stepwise approach to tackle this problem: 1) primary needle trajectories were generated using the classical Hough transform algorithm; 2) to ensure clinical feasibility and collinearity between seeds and needles, an optimization-based refinement method was applied to adjust initial needle placements while satisfying both hard and soft constraints derived from surgical and robotic considerations.

### 2.1 Primary needle placement generation

To identify potential needle trajectories passing through clustered seeds, a 3D line detection method based on the Hough transform was employed. The classical Hough transform converts a geometric entity (such as a point or line) in the Cartesian coordinate system into a parametric representation in a transformed “voting space”. Each seed position contributes a “vote” to all possible line parameters that could pass through it. Peaks in this voting space correspond to lines supported by multiple nearby seeds, representing potential needle directions.

While the traditional Hough transform operates in two dimensions for line detection in images, in this work, it was extended to three-dimensional seed coordinates using methods provided by Dalitz (28). The 3D Hough space was parameterized by a direction vector $\vec{b}$ describing the insertion orientation within the body and an anchor point $A(x^{\prime },y^{\prime })$, which lies on a plane passing through the origin and orthogonal to $\vec{b}$. The coordinates $\left(x^{\prime },y^{\prime }\right)$ provide a two-dimensional parameterization of this plane and uniquely define the spatial position of the corresponding line. Each seed point P(x,y,z) in the Cartesian space was projected to a family of possible $\left(x^{\prime },y^{\prime },\vec{b}\right)$ combinations in the Hough space using the forward transform (Equations 1, 2). The cumulative voting values were then used to identify dominant lines that best align multiple seed points, forming initial needle candidates.

(1) $$\vec{b}=(\begin{array}{c} b_{x}\\ b_{y}\\ b_{z} \end{array})=(\begin{array}{c} \text{cos}\varphi \text{cos}\theta \\ \text{sin}\varphi \text{cos}\theta \\ \text{sin}\theta \end{array})$$

(2) $$\left\{ \begin{array}{c} x^{\prime }=(1-\frac{b_{x}^{2}}{1+b_{z}})p_{x}-(\frac{b_{x}b_{y}}{1+b_{z}})p_{y}-b_{x}p_{z}\\ y'=-(\frac{b_{x}b_{y}}{1+b_{z}})p_{x}-(1-\frac{b_{y}^{2}}{1+b_{z}})p_{y}-b_{y}p_{z} \end{array}\right.$$

The cumulative voting value represents the number of points that are approximately co-linear. Then, the inverse transformation (Equation 3) is used to obtain the Cartesian space representation of the anchor point. A two-point representation is adopted to facilitate constraint modeling. Specifically, the needle is parameterized by a shaft reference point *P_S_* and a needle tip point *P_T_*. The scalar parameter *t* denotes the minimal distance along the needle direction vector from the current querying point *P_q_* to the anchor point. It is computed as in Equation 4. The needle tip point *P_T_* is then obtained as in Equation 5. The needle shaft point *P_S_* is defined by extending the needle by a fixed offset *t_l_*, which corresponds to the physical length of the needle (Equation 6).

(3) $$\begin{array}{r} \vec{P}=x^{\prime }(\begin{array}{c} 1-\frac{b_{x}^{2}}{1+b_{z}}\\ -\frac{b_{x}b_{y}}{1+b_{z}}\\ -b_{x} \end{array})+y^{\prime }(\begin{array}{c} -\frac{b_{x}b_{y}}{1+b_{z}}\\ 1-\frac{b_{y}^{2}}{1+b_{z}}\\ -b_{y} \end{array}) \end{array}$$

(4) $$t=\vec{b}\cdot (\vec{P_{q}}-\vec{P})$$

(5) $$P_{T}=\vec{P}+t\vec{b}$$

(6) $$P_{S}=\vec{P}+(t+t_{l})\vec{b}$$

Conceptually, the Hough-based grouping replaces the manual visual alignment of seeds with an automatic “voting” mechanism, enabling consistent and reproducible identification of multi-seed trajectories before robotic adjustment.

The degree of discretization in the parameter space and the chosen partitioning strategy directly determine its ability to distinguish between lines. The computation time of the Hough transform is not significantly correlated with the number of points in the seed cloud but rather with the number of discretization units. In this study, icosahedral subdivision was used for angular discretization in 3D space. Under six levels of iteration, the angular resolution reached 1.3°, and the spatial resolution was set to 0.2 mm. These parameters provided a good balance between computation efficiency and spatial accuracy, resulting in a per-case computation time of less than 2 sec and a maximum deviation of 0.08 mm between the identified line and the actual seed positions. The deviation is sufficiently small that it does not affect the subsequent optimization steps.

### 2.2 Optimization-based needle placement refinement

The primary needle placements obtained from the Hough transform provide an initial geometric reference for potential insertion trajectories. However, these preliminary paths may still intersect anatomical obstacles or violate robotic workspace limits. To ensure clinical feasibility, an optimization-based refinement step was introduced to locally adjust each needle trajectory while maintaining alignment with its associated seed group.

The goal of this refinement is to minimize deviations between the planned seeds and their corresponding optimized needle axes, subject to both surgical and robotic constraints. The adjustment is performed within a limited spatial neighborhood to preserve the overall geometry of the Hough-derived trajectory. For each needle, the shaft point *P_S_* is allowed to move within a cubic neighborhood $\left(x_{0}\pm r,y_{0}\pm r,z_{0}\pm r\right)$ around its initial position $P_{S}^{0}(x_{0},y_{0},z_{0})$, while the tip *P_T_* remains fixed. The optimization minimizes the sum of perpendicular distances from each seed to its corresponding needle axis, as expressed in Equations 7, 8.

(7) $$e_{ij}=\|\frac{\left(P_{ij}-P_{S})\;\times \;(P_{ij}-P_{T}\right)}{∥P_{S}-P_{T}{∥}_{2}}{\|}_{2}$$

(8) $$\text{err}=\sum_{i}^{m}\sum_{j}^{n}e_{j}$$

This formulation ensures that each needle passes as close as possible to all assigned seed dwell positions while maintaining mechanical and anatomical feasibility. To achieve this, two categories of constraints are introduced—*hard* and *soft* constraints. Hard constraints represent non-negotiable safety or mechanical limits, while soft constraints encode desirable geometric or clinical conditions that may be relaxed slightly during optimization.

#### 2.2.1 Hard constraints

1. Needle accessibility constraint: The planned depth of the needle placement within the body should not exceed its maximum length, as shown in Equation 9.

(9) $$∥P_{T}-P_{S}{∥}_{2}\leq L_{max}$$

#### 2.2.2 Soft constraints

1. Obstacle constraint: The needle must maintain a safety margin from the obstacle. Irregularly shaped internal obstacles are enveloped by multiple spheres with equal and adaptive radius, allowing the obstacle boundary and distance to be represented by the sphere radius, as shown in Equation 10.

(10) $$\forall \|\frac{\left(\text{center }-\;P_{S})\times (\text{center }-\;P_{T}\right)}{∥P_{S}-P_{T}{∥}_{2}}{\|}_{2}\geq \text{radius}$$

2. Robot end-effector workspace constraint: In multi-needle brachytherapy procedures, needles are densely packed, and ample operation space must be reserved for the gripper when using a robot for needle puncture operations, as shown in Equation 11. We utilized a two-finger gripper for picking and puncturing with a working space radius $d_{g}=20$ mm.

(11) $$∥P_{T1}-P_{T2}{∥}_{2}\geq d_{g}$$

Needle stability constraint: To ensure the stability of the needle, the needle must maintain enough depth, as shown in Equation 12.

(12) $$∥P_{T}-P_{S}{∥}_{2}\geq L_{\text{min}}$$

Apart from the soft constraints mentioned earlier, a local modification also requires an extra constraint, the needle length constraint $C_{l}$, that is, the length of the puncture needle that remains fixed, as shown in Equation 13.

(13) $$∥P_{S}-P_{T}{∥}_{2}-C_{l}=0$$

The optimization problem defined by these objectives and constraints was solved using the Sequential Least Squares Programming (SLSQP) solver implemented in SciPy. This method efficiently handles non-linear equality and inequality constraints, providing smooth convergence for multi-needle configurations.

Conceptually, this stage ensures that all optimized needle trajectories satisfy anatomical safety, mechanical feasibility, and robotic workspace requirements by adjusting the needle tip positions within a cubic search space.

### 2.3 Simulation

Before generating feasible needle trajectories, an initial set of planned seed positions was created using a geometry-based procedure. Random tumor shapes were generated using a multi-sphere aggregation approach. Each tumor was constructed by placing several spheres of equal radius at selected positions within the target volume. The centers of these spheres were used as the initial planned seed locations. The union of all spheres was then enveloped by a smooth outer boundary, producing continuous, irregular tumor-like geometries suitable for needle placement planning. The number and radii of the spheres were empirically selected to produce realistic tumor volumes while preserving the prescribed dose distribution. Each tumor shape contained between 20 and 30 seeds, reflecting typical clinical scenarios. The spheres’ centers of generated tumors were then used as initial seed input for the needle placement planning.

Twenty randomly shaped tumors were generated to simulate hepatic lesions. Needle path planning and subsequent evaluation were performed accordingly. Iodine-125 seeds (model 6711) with an activity of 0.5 mCi were used in the simulations.

The abdomen dataset, organ segmentation models, and needle placement modeling functions were obtained from a prior study by Yamada (29). These modeling functions were used consistently with the present implementation. Dose computation followed the TG-43 formalism, and slicerRT (30) was used for dose comparison and generation of DVH plots. Due to the finite resolution (approximately 3–4 mm in XY directions; 1.5 mm in Z direction) of the three-dimensional dose grid, extremely high dose regions in the immediate vicinity of radioactive sources were not fully resolved, resulting in a numerical truncation of the high-dose tail of the Planning Target Volume (PTV) DVH. The planning algorithm was implemented using Python, with 3D reconstruction and visualization handled using 3D Slicer (31). The experiments were performed on a system equipped with an Intel^®^ Core™ i7-7700HQ CPU @ 2.80 GHz processor.

A needle was considered feasible if it satisfied all geometric hard constraints and soft constraints described in Section 2.2. The seed adjustment distance was defined as the 3D Euclidean displacement between the nominal seed positions and their optimized locations. Gamma analysis was performed to compare the dose distributions before and after the needle trajectory optimization using a distance criterion of 2 mm and a dose criterion of 2% (global gamma normalization).

### 2.4 Phantom experiment

**Figure 2:**
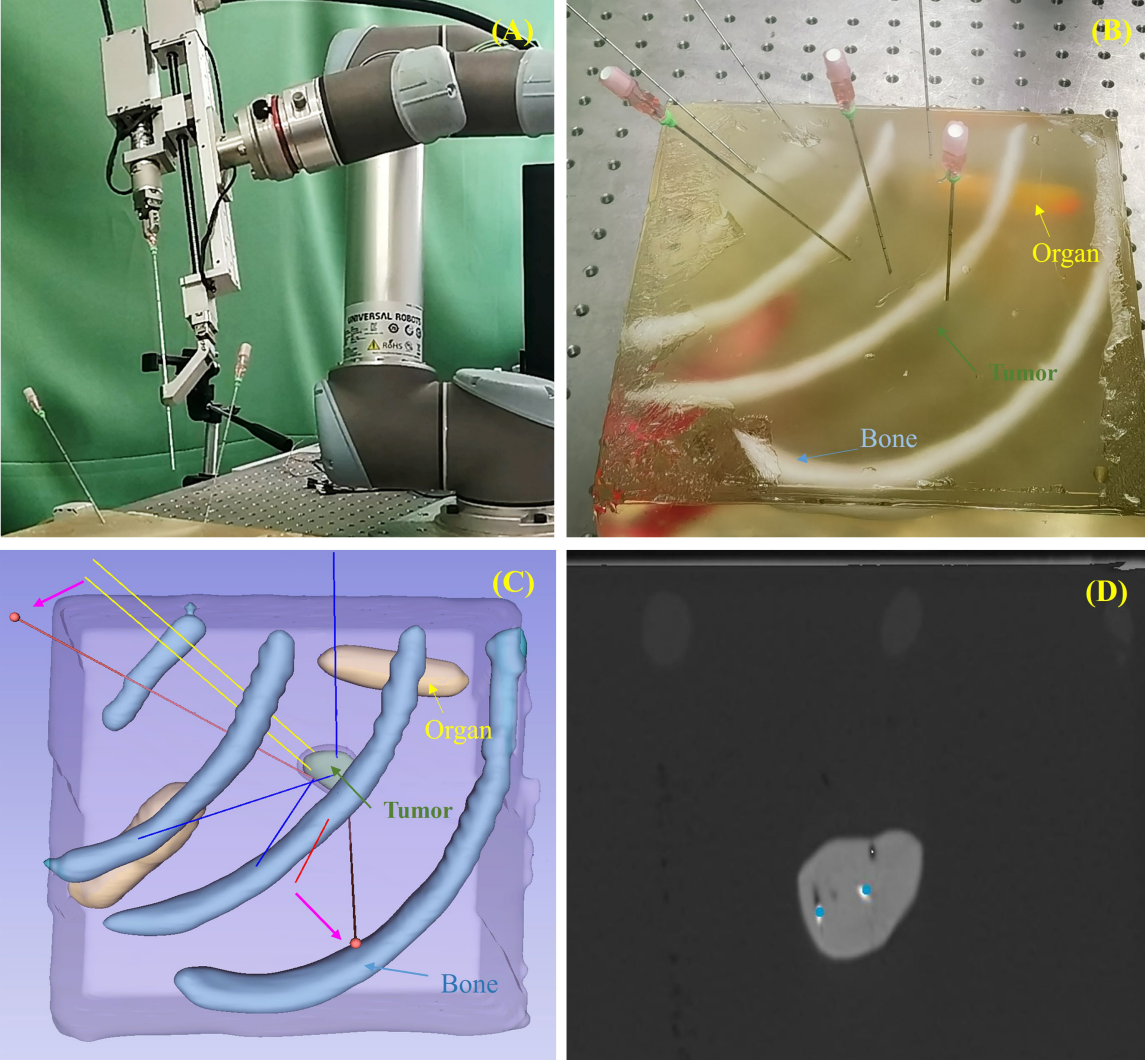
Phantom experiment. **(A)** Experimental platform including the robotic arm, puncture device, and silicone phantom. **(B)** Needle placement planning results; pink arrows indicate refinement of needles that were too close to neighboring needles or intersected with bones. **(C)** Silicone phantom after robotic needle insertion. **(D)** Post-implant CT image of implanted seeds; preplanned seed dwell positions are shown as blue dots.

Phantom experiments were conducted to validate the practical feasibility and accuracy of the proposed needle placement planning method under robotic operation conditions. Our experimental platform is shown in **Figure 2A**, which includes a UR5 robotic arm (Universal Robots A/S, Odense, Denmark) equipped with a custom puncture end-effector and a silicone phantom simulating human anatomy, including artificial bones, organs, and tumor-like structures. Five needle insertion experiments were conducted. Each phantom was first scanned using CT (slice thickness, 0.3 mm; field of view, 144 * 124 mm) to obtain preoperative imaging data. Based on the three-dimensional reconstructions, the geometric shape of the tumor was determined. Following the inverse geometry-based initialization strategy described in Section 2.3, a sphere-based partitioning algorithm was employed to determine the initial seed positions, and the target seed dwell positions were predefined. Needle placement planning was then performed according to the methods described in Sections 2.1 and 2.2.

The UR5 robotic arm, equipped with a custom puncture end-effector capable of rotation and linear feed, executed the planned trajectories. The UR5 provides a nominal repeatability of ±0.1 mm, which is sufficient for the needle placement task considered in this study. Tool calibration and image-to-robot registration were performed following our previously published method (32–34). Specifically, the tool calibration error was within ±0.05-mm accuracy, and the image-to-robot registration achieved an average error below 0.5 mm, with high repeatability across repeated trials. After trajectory execution, seeds were manually inserted, and post-implant CT scans were acquired for evaluation.

Implanted seed positions were determined using thresholding followed by Gaussian mixture regression (GMR) to identify the seed centers. To evaluate the implantation accuracy, the spatial errors between the implanted seed positions and the planned seed locations were quantified. Axial errors were defined along the needle axis, with positive values indicating displacement toward the needle tip and negative values indicating displacement in the opposite direction. Radial errors were measured in the direction perpendicular to the needle axis.

### 2.5 Comparison study

We compared the proposed method with a conventional coplanar template approach on five cases consistent with Section 2.4, yielding a total of 10 cases. The coplanar template layout was generated manually by an experienced clinician following geometric heuristics (surface-normal insertion and parallel coplanar constraints). This manually designed baseline represents a commonly used clinical strategy and was used for comparison with the proposed automatic method.

To ensure a fair comparison, both planning approaches employed the same seed model and the same total number of seeds; only the spatial distribution of the seeds differed between the two methods. The resulting dose distributions were evaluated and compared using DVHs, dose field cross-sectional visualizations, and quantitative dosimetric metrics including the *D*_mean_, *V*_150_, and the conformity index (CI). The conformity index was computed using Paddick’s formulation, defined as shown in Equation 14.

(14) $$\text{CI}=\frac{{\left(V_{\text{PTV}\cap \text{PIV}}\right)}^{2}}{V_{\text{PTV}}\;\cdot \;V_{\text{PIV}}},$$

where $V_{\text{PTV}}$ denotes the target volume, $V_{\text{PIV}}$ is the volume enclosed by the prescription isodose, and $V_{\text{PTV}\cap \text{PIV}}$ represents their intersection.

## 3 Results

### 3.1 Simulation results

**Figure 3:**
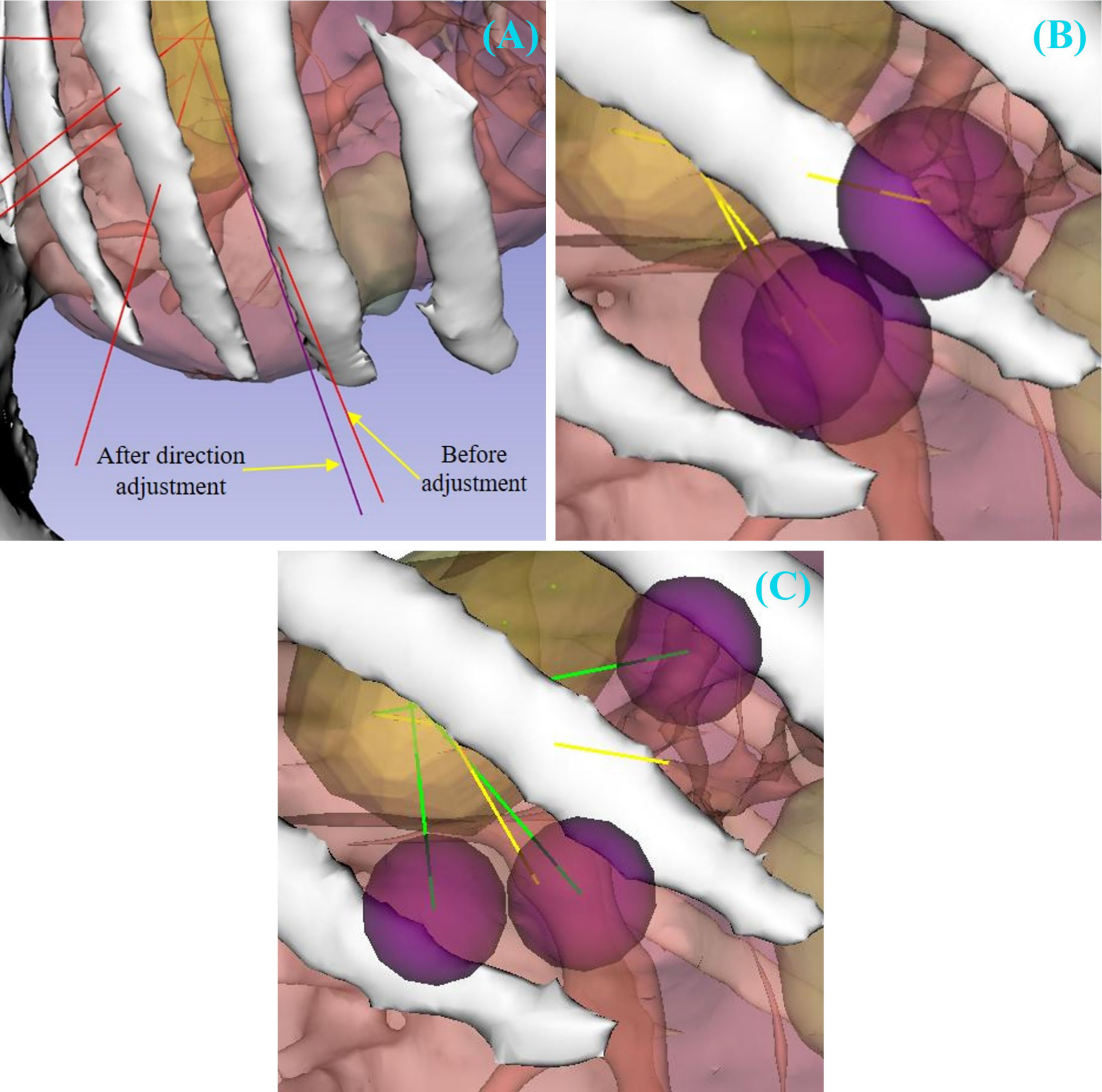
Representative needle refinement results under different constraints. **(A)** Needle direction adjustment under obstacle constraints. Red lines depict needle placement before adjustment, and purple lines depict needle placement after adjustment. **(B)** End-effector operational workspace before directional adjustment. Purple spheres intersect, indicating potential collisions during grasping. **(C)** After directional adjustment, the operational spheres no longer intersect, ensuring sufficient workspace for the needle shaft.

As summarized in **Table 1**, across the 20 cases, the proposed method generated feasible needle sets with high reliability. Reliability was assessed based on seed reachability, the maximum adjustment distance applied to seeds, and the overall gamma pass rate, rather than the absolute number of needles generated per case. On average, 8.2 ± 2.1 feasible needles were produced per case, with a mean maximum seed adjustment distance of 0.6 ± 0.3 mm. The average gamma pass rate was 97.3% ± 2.7%. Not all seeds were reachable in every case: for example, Case C7 contained two missed seeds, consistent with **Table 1**. **Figure 3** illustrates examples of optimization results under different constraints. A case was considered successful if all seeds were reachable, or if the displacement of all reachable seeds remained below 1.5 mm, resulting in an overall simulation success rate of 95%.

**Table 1:** Summary of planning accuracy and needle modifications.

Case	Total seed number	Missed seeds	Needles	Total modified needles	Max seed adjustment (mm)	Gamma (%)
C1	25	–	6	1	0.33	98.2
C2	25	–	10	3	0.41	97.5
C3	25	–	6	1	0.59	96.1
C4	25	–	9	1	0.34	98.0
C5	25	–	6	2	1.20	92.5
C6	25	–	11	5	1.36	91.9
C7	25	2	–	–	–	–
C8	25	–	6	5	0.58	96.3
C9	25	–	10	5	0.86	95.9
C10	25	–	11	4	0.81	94.8
C11	30	–	6	2	0.64	95.6
C12	30	–	7	2	0.72	93.7
C13	30	–	6	1	0.35	97.8
C14	30	–	9	4	0.47	96.9
C15	30	–	10	5	0.33	97.5
C16	30	–	11	3	0.31	98.4
C17	30	–	12	3	0.49	97.1
C18	30	–	8	3	1.24	92.6
C19	30	–	8	2	0.87	94.8
C20	30	–	6	2	0.61	95.4
Mean ± SD	–	–	8.2 ± 2.1	–	0.6 ± 0.3	97.3 ± 2.7

“Total seed number” indicates the total number of seeds planned for the case. “Missed seeds” refers to seeds that become unreachable after optimization due to violations of geometric hard constraints or because their corresponding needle paths fall outside the robot’s workspace. “Needles” is the total number of needles used per case. “Total modified needles” indicates needles whose planned trajectories required adjustment during optimization. “Max seed adjustment” represents the maximum displacement applied to any seed during the trajectory optimization step. The gamma index was computed between the planned and optimized dose distributions using distance criteria of 2 mm and dose criteria of 2%, respectively.

**Figure 4:**
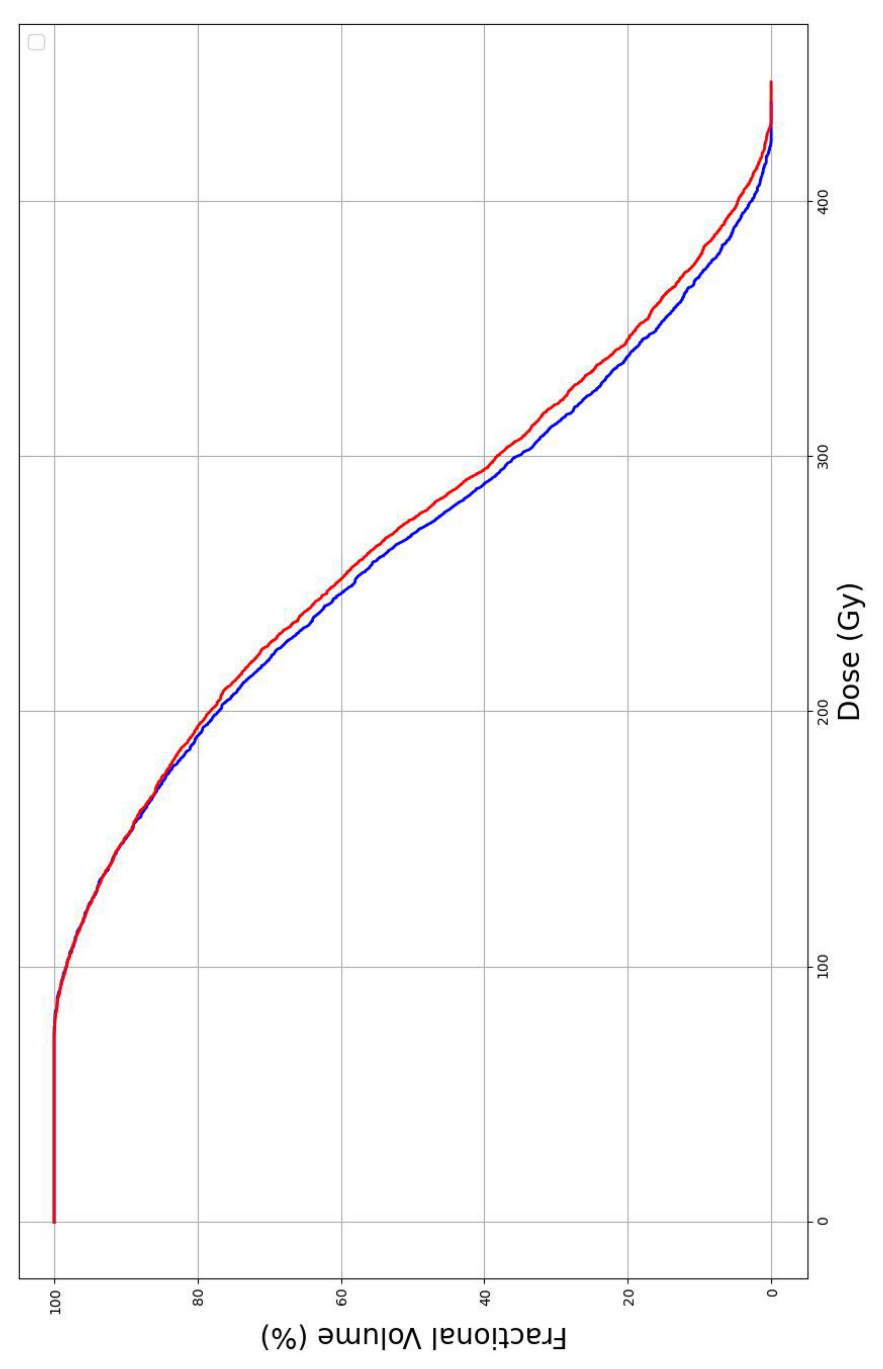
Representative DVH curves before and after needle trajectory optimization for a single representative case. Blue lines denote the dose distribution generated from initial seed positions, whereas red lines denote the dose distribution after needle placement optimization. Note that the PTV DVH maximum is truncated due to the discrete dose grid used in the calculations. DVH, dose–volume histogram.

In addition to Gamma analysis, DVH plots were generated to compare dose distributions before and after needle trajectory optimization or a single representative case, as in **Figure 4**. The DVH curves showed minimal differences, indicating that the optimization process preserved the target dose distribution. Primary needle-path generation (Hough transform) required less than 1 sec per case, while trajectory optimization required an average of 43 sec per needle.

### 3.2 Phantom experiment

**Figure 2B** shows the planned needle placement for one representative case, while **Figure 2C** presents the actual needle insertion performed by the robot; the two results are consistent. **Figure 2D** shows the corresponding post-implant CT scan.

**Figure 5:**
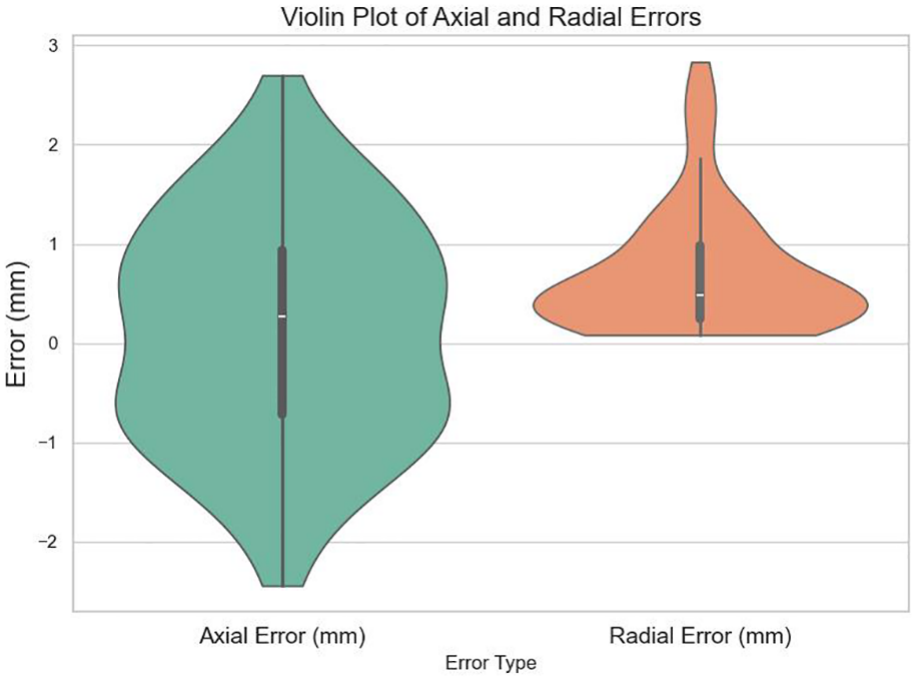
Violin plot of silicone phantom seed implantation error distribution. Axial error is defined as the displacement of the implanted seed along the planned needle direction, where the positive direction points toward the needle tip and the negative direction points toward the needle shaft. Radial error refers to the perpendicular deviation of the seed position from the planned needle direction.

Across all insertions, the mean deviation of axial and radial errors was 0.11 mm and 0.713 mm, respectively. Axial deviations ranged from a maximum of +2.69 mm in the needle insertion direction to −2.44 mm in the opposite direction. Of the seeds, 63.3% exhibited an axial error below 1 mm, and 96.6% were within 2 mm. Radial deviations reached a maximum of 2.37 mm, with 76.6% of the seeds showing radial errors below 1 mm and 93.3% within 2 mm. The deviations of all seeds were analyzed along the axial and radial directions, and the results were summarized and visualized as violin plots in **Figure 5**.

### 3.3 Comparison study

**Figure 6:**
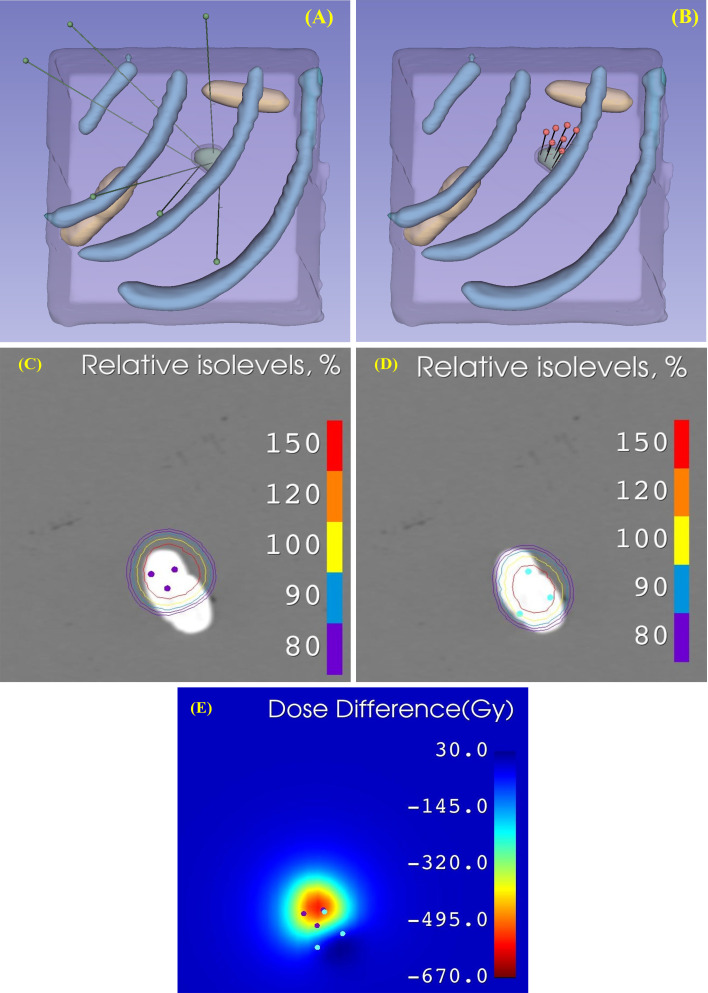
Comparison of two seed distribution styles. **(A)** Geometry-based distribution planning result. **(B)** Coplanar template-based manual planning result. **(C)** Isolevel diagram of Case **(A)** Blue dots represent seed dwell positions in geometry-based planning. **(D)** Isolevel diagram of case **(B)**. Red dots represent seed dwell positions in coplanar template-based planning. **(E)** Coronal dose difference map (geometry-based minus coplanar). Blue dots indicate geometry-based seed positions, while purple dots indicate coplanar seed positions.

**Figure 6** is a representative instance, where the two planning approaches are shown in panels A and B, the isolevel plots of dose distributions are shown in panels C and D, and panel E presents the cross-sectional view of the difference in dose distributions.

**Figure 7:**
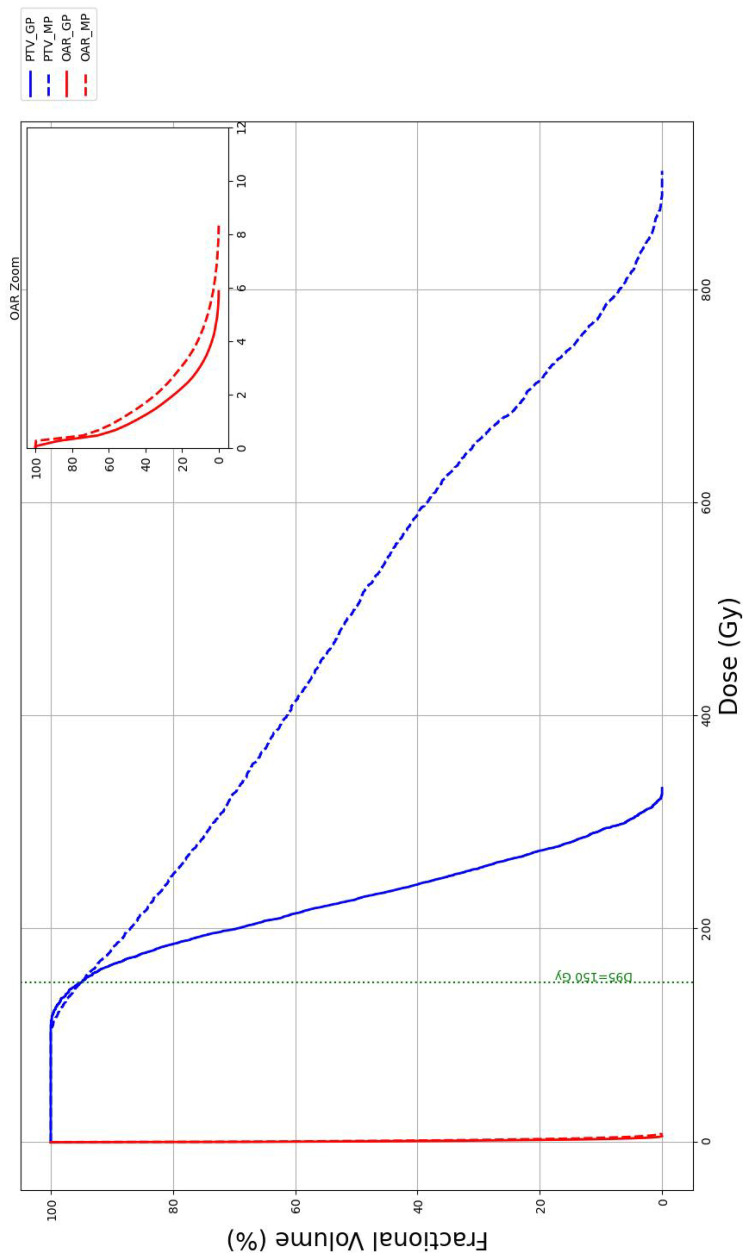
Representative DVH curves for a single representative case. Solid lines denote the geometry-based planning method, whereas dashed lines denote the coplanar planning method. Blue curves indicate the PTV, and red curves indicate the nearest OAR. The inset provides a zoomed-in view of the OAR DVH, with an expanded dose range along the x-axis while keeping the y-axis scale unchanged. Note that the PTV DVH maximum is truncated due to the discrete dose grid used in the calculations. DVH, dose–volume histogram; OAR, organ at risk.

The DVH result for a single representative case is shown in **Figure 7**, and similar trends were observed across all cases. Solid lines represent TPS planned (group TP) dose distributions from our method, while dashed lines correspond to the coplanar template manual planning approach (group MP). Blue curves indicate the PTV, and red curves indicate the organ at risk (OAR). The inset provides a zoomed-in view of the OAR DVH, with an expanded dose range along the x-axis while keeping the y-axis scale unchanged. All DVHs were normalized such that the prescribed dose was equal across plans, ensuring fair comparison. The DVH curves produced by our dose-driven needle planning demonstrate a sharper decline beyond the prescription dose.

The dosimetric comparison between the proposed and manual plans across 10 cases, as summarized in **Table 2**, showed that the proposed method improved dose homogeneity, with lower high-dose volumes (*V*_150_ = 56.9% *vs*. 72.9%, *p* = 0.002; *V*_200_ = 22.2% *vs*. 43.2%, *p* = 0.012) and reduced mean doses (*D*_mean_ = 247.6 ± 25.7 Gy *vs*. 320.3 ± 76.1 Gy, *p* = 0.019). Additionally, the proposed method achieved higher CI values (CI = 0.67 ± 0.1 *vs*. 0.45 ± 0.17, *p* = 0.011). All *p*-values were computed using a paired two-sided t-test.

**Table 2:** Dosimetric comparison between the proposed and manual plans across 10 cases.

Case	$V_{150}$ (%)	$V_{200}$ (%)	$D_{90}$ (Gy)	$D_{mean}$ (Gy)	CI
C1	$\frac{52.6}{83.5}$	$\frac{6.1}{73.4}$	$\frac{319.2}{868.7}$	$\frac{228.6}{490.6}$	$\frac{0.59}{0.26}$
C2	$\frac{33.7}{56.8}$	$\frac{6.3}{16.8}$	$\frac{265.0}{329.1}$	$\frac{207.9}{231.6}$	$\frac{0.68}{0.51}$
C3	$\frac{60.3}{73.1}$	$\frac{25.3}{38.1}$	$\frac{366.7}{485.7}$	$\frac{249.0}{283.6}$	$\frac{0.56}{0.37}$
C4	$\frac{59.3}{73.7}$	$\frac{16.0}{47.5}$	$\frac{343.8}{551.3}$	$\frac{239.3}{304.7}$	$\frac{0.83}{0.63}$
C5	$\frac{56.7}{73.4}$	$\frac{26.5}{48.6}$	$\frac{445.3}{734.4}$	$\frac{253.2}{331.2}$	$\frac{0.69}{0.47}$
C6	$\frac{76.4}{78.6}$	$\frac{50.5}{58.9}$	$\frac{402.4}{712.0}$	$\frac{245.8}{320.2}$	$\frac{0.64}{0.46}$
C8	$\frac{58.5}{88.1}$	$\frac{23.4}{45.9}$	$\frac{565.1}{485.4}$	$\frac{258.4}{312.6}$	$\frac{0.70}{0.41}$
C9	$\frac{59.3}{57.9}$	$\frac{33.8}{50.2}$	$\frac{361.5}{358.8}$	$\frac{243.0}{244.5}$	$\frac{0.65}{0.48}$
C10	$\frac{62.8}{79.3}$	$\frac{17.9}{20.1}$	$\frac{423.1}{517.2}$	$\frac{269.1}{345.8}$	$\frac{0.71}{0.44}$
Mean ± SD	$\frac{56.9\;\pm \;11.0}{72.9\;\pm \;10.4}$	$\frac{22.2\;\pm \;14.9}{43.2\;\pm \;19.9}$	$\frac{396.4\;\pm \;93.1}{590.1\;\pm \;192.6}$	$\frac{247.6\;\pm \;25.7}{320.3\;\pm \;76.1}$	$\frac{0.67\;\pm \;0.08}{0.45\;\pm \;0.17}$
*p*-Value	0.002	0.012	0.016	0.019	0.001

Each cell shows the value for proposed (top) and manual (bottom) plans for each metric.

CI, conformity index.

## 4 Discussion

The results of this study demonstrate that the proposed needle placement framework can reliably translate planned seed positions into feasible, robot-executable trajectories while maintaining clinically relevant dosimetric quality. The trajectory optimization effectively preserves the intended dose distribution, as supported by Gamma analysis and DVH comparisons, which show maintained target coverage and respect for OAR constraints. Compared with conventional manual planning, our method improves dose shaping around the PTV and enhances conformity, suggesting better organ sparing and more homogeneous dose distribution. Simulation experiments indicate minimal deviation from the nominal seed position, with an average seed adjustment distance of 0.6 ± 0.3 mm and a simulation success rate of 95%, suggesting that trajectory optimization preserves the intended dose distribution. Gamma analysis confirms that the planned dose distributions are maintained, and DVH comparisons show that target coverage is preserved while respecting OAR constraints. A comparison study between our approach and conventional manual planning shows that dose homogeneity within the PTV is improved, reflected by lower high-dose volumes and reduced mean doses, and the method achieves improved dose shaping around the PTV, reflected by improved CI, enhancing organ sparing.

Phantom experiments confirm that robotic-assisted needle insertions achieve high spatial accuracy, primarily reflecting the intrinsic precision of the UR5 robot, including calibration and repeatability verified under controlled conditions. These findings highlight the feasibility of integrating robotic brachytherapy with planned seed distributions to achieve precise, clinically relevant implantations, potentially reducing intraoperative adjustments, improving reproducibility, and shortening procedure time. Several limitations should be acknowledged. First, the current framework assumes static tumor and organ geometry; in clinical scenarios such as lung brachytherapy, respiratory motion may affect dosimetric accuracy. Incorporating motion prediction and real-time compensation would be necessary to address this issue. Second, trajectory optimization was performed on manually generated seed distributions without full TPS-based dose re-optimization. Although the observed seed deviations were generally below 2 mm, future work should explore combining dose optimization with robotic feasibility to further enhance clinical relevance. Third, local infeasibility may occur when multiple constraints—such as end-effector workspace limitations and obstacle avoidance—are simultaneously active. In such cases, enlarging the optimizer’s search region often helps recover feasible solutions. In our experiments, approximately 10% of the cases required this strategy. While the proposed adjustment strategies mitigate some infeasible placements, suboptimal initial seed distributions may still limit performance. Finally, the number of phantom experiments was limited, and OAR models were manually constructed rather than strictly following anatomical contours, which may restrict generalizability.

Overall, this framework provides a foundation for extending robotic-assisted brachytherapy to anatomically complex regions, such as hepatic or thoracic tumors, and motivates future work incorporating patient-specific motion compensation and combined dose–robot optimization. Potential directions include integrating robotic performance metrics—such as manipulability, collision probability, and force limits—into the cost function to enhance procedural safety and employing learning-based models for seed distribution and obstacle avoidance to enable a fully automated, intelligent robotic brachytherapy system.

## 5 Conclusion

This study presented a post-TPS optimization framework for robotic-assisted brachytherapy that connects dose-optimized seed planning with robotically feasible needle trajectories. By integrating 3D Hough transform-based seed grouping and optimization-based trajectory refinement, the method ensures obstacle-free, clinically executable, and robot-safe needle paths.

Simulation and phantom experiments demonstrated that the framework achieves sub-millimeter placement accuracy while preserving dose quality, confirming its feasibility for translating virtual treatment plans into physical robotic procedures.

The proposed framework effectively bridges the gap between traditional treatment planning systems and robotic execution in brachytherapy, offering a practical pathway toward intelligent, image-guided, and automated interventional oncology. Future work will extend the optimization model to include robotic performance indices and learning-based planning strategies, paving the way for fully autonomous robotic brachytherapy and broader applications in precision cancer treatment.

## Data Availability

The original contributions presented in the study are included in the article/supplementary material. Further inquiries can be directed to the corresponding author.
